# Growth Velocity in South Indian Children Between Three and 18 Years of Age

**DOI:** 10.7759/cureus.50865

**Published:** 2023-12-20

**Authors:** Gayatri Sabinkar, Babulal Sabinkar, Vijaya Sarathi, Dileep K Kumar

**Affiliations:** 1 Endocrinology and Metabolism, All India Institute of Medical Sciences, Bibinagar, Hyderabad, IND; 2 Pulmonary Medicine, Nimra Institute of Medical Sciences, Vijayawada, IND; 3 Endocrinology and Diabetes, Vydehi Institute of Medical Sciences and Research Centre, Bangalore, IND; 4 Endocrinology, Narayana Medical College and Hospital, Nellore, IND

**Keywords:** growth monitoring, short stature (ss), south indian, height velocity, growth velocity charts

## Abstract

Objective

Height velocity is a crucial anthropometric parameter for the evaluation of mild- or recent-onset short stature; however, there is no data on height velocity in South Indian children. We undertook this study to establish the normative data.

Methods

This prospective longitudinal study included 3327 apparently healthy children aged three to 18 years from government and private schools of Krishna district, Andhra Pradesh. Height and weight were measured at baseline and three-monthly intervals for one year (October 2018 to October 2019).

Results

Age- and sex-specific height velocity percentiles were generated. The data was available in 1627 boys and 1700 girls. The mean peak height velocity (PHV) was 7.18±2.56 cm in boys observed at 12-12.9 years and 5.8±2.56 cm in girls at 10-10.9 years.

Conclusion

Normative height velocity data for South Indian children has been presented.

## Introduction

Growth references are the most valuable and commonly used tools for assessing the general well-being of a child, group of children, and their communities, and for tracking progress in reaching health-related and other broader goals of social equity [1]. Longitudinal rather than cross-sectional growth references, despite the logistic limitations, are believed to be a better representative of an individual’s growth pattern. Low height velocity (HV) alone (<−2 standard deviation score (SDS) over one year or <−1.5 SDS over two years), even in the absence of apparent short stature (height SDS <−2) is a criterion for the evaluation of growth disorders [2]. Thus, examining growth velocity may help in the early identification and treatment of growth failure [1,3-5].

Worldwide, the positive secular trend has led to increased height and weight during childhood and also a downward trend in the age of menarche, with some differences in various countries [6]. Over the last two decades, this trend towards earlier menarche has declined in most developed countries since socioeconomic and nutritional conditions have reached optimal levels [7]. The secular trends continue to show a positive change in developing and underdeveloped countries. Hence, it has been recommended that these references be updated regularly [8]. The WHO also recommends that every country update its growth references every decade.

The use of WHO reference charts is likely to over-diagnose stunting or underweight in developing countries, and many authors from many countries have expressed concerns regarding this. Height velocity charts are already available for a few countries [9-11]. Data from Asian countries such as Japan, China, and Saudi Arabia have shown that the growth of older non-European children is different, especially after puberty, with a relatively attenuated pubertal growth spurt [12,13]. The patterns of weight and height changes in Indian children are also different from the rest of the world due to various factors like genetics, diet, lifestyle, health conditions, physical activities, and environment. Hence, there is an imminent need to generate good-quality HV data for our country [14]. This demands a long follow-up of years involving a sufficiently large, healthy, ethnically and geographically diverse population to capture the normal variability in height [15]. Though there have been recent publications of height velocity data from North India [16] and West India [17], there has not been any publication of height velocity data of South Indian children in the past decade.

This article was previously presented as a poster at the 2022 International Congress of Endocrinology and won the International Society of Endocrinology (ISE) abstract award.

## Materials and methods

This prospective, longitudinal study recruited children aged three to 18 years from rural and urban preschools, schools, and colleges in the Nandamuri Taraka Ramarao (NTR) district of Andhra Pradesh. The study was approved by the institutional ethics committee of Narayana Medical College, Nellore, Andhra Pradesh (reference letter dt 30-04-2018). Assent of all children aged seven years and older and written consent from the legal guardians of all children were obtained.

The date of birth information of all participants was noted. Anthropometric measurements (weight and height) of children were recorded at baseline and three-monthly intervals for one year between October 2018 and October 2019, with the help of trained volunteers under the supervision of GS and BS. Standard anthropometric measurement techniques, as described by the WHO, were adopted [18]. Children with known serious or chronic medical illnesses that interfere with growth velocity were excluded from the study.

All the data was entered and then analyzed using SPSS for Windows 22.0 (IBM Corp., Armonk, New York). Categorical variables were described as frequency or percentages, whereas continuous parameters were expressed as mean±SD. Differences between the two groups were compared by student T-test or chi-square test as appropriate. For all analyses, a P-value of <0.05 was considered statistically significant. Height velocity charts were prepared.

## Results

**Figure 1:**
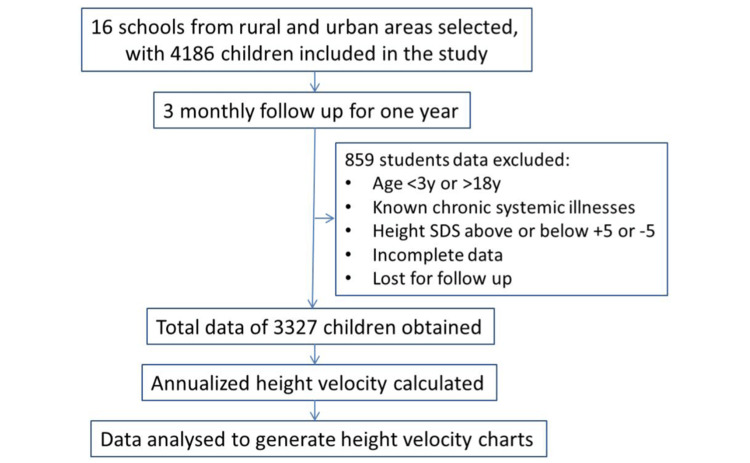
Flowchart depicting the recruitment of children for the analyses.

In the NTR district, out of all the schools that were approached for the study, the management authorities of 16 preschools, schools, and colleges consented to the study. Out of all the students admitted to these educational institutions, depending on the number of students present on that day, 4186 children were recruited at the baseline visit. By the end of one year, data related to 3327 students was included in the final analyses, as shown in Figure 1. Data pertaining to 859 students was eliminated because of the presence of obvious chronic illnesses (like type 1 diabetes, epilepsy, or chronic diarrhea) or height SDS above +5 SD or below −5 SD was eliminated as obvious outliers [19]. Also included in this category were the students who were lost to follow-up due to changing or discontinuation of school (especially those in upper kindergarten, classes 10 and 12) or absence during one of the follow-up visits and those who were less than three years or more than 18 years at the start of the study.

Out of the 3327 children analyzed, 1627 (48.9%) were boys, and 1700 (51.1%) were girls. Among our subjects, 676 boys (46.36%) and 782 girls (53.64%) were from rural areas, and 951 boys (50.88%) and 918 girls (49.12%) were from urban areas. Height velocity (HV) charts can be generated based on the available data. The peak height velocity (PHV) attained was 5.80±2.56 cm at 10-11 years of age in girls and 7.18±2.56 cm at 12-13 years of age in boys (Table 1).

**Table 1:** Mean height velocity in boys and girls at different ages. HV: height velocity.

Age in years	Number of boys	HV in cm (mean±SD)	Number of girls	HV in cm (mean±SD)
3-4	22	6.47±1.85	24	7.96±2.60
4-5	20	6.13±1.74	29	6.60±2.17
5-6	32	5.79±1.38	39	6.01±2.09
6-7	71	5.46±1.15	73	5.38±1.68
7-8	87	5.03±1.14	77	5.07±1.49
8-9	98	4.95±1.71	101	5.41±1.95
9-10	93	5.23±1.50	89	5.21±2.07
10-11	103	5.86±1.80	134	5.80±2.56
11-12	136	6.13±1.75	172	5.24±2.78
12-13	168	7.18±2.56	185	3.91±2.61
13-14	220	6.40±2.40	228	3.26±2.62
14-15	212	4.43±2.64	238	2.63±2.29
15-16	193	2.75±2.28	173	2.33±1.70
16-17	135	2.30±2.04	116	1.68±1.36
17-18	37	1.86±1.29	22	0.66±0.43

The peak median height velocity was 5.2 cm in girls and 6.5 cm in boys (Table 2). The height velocity of girls and boys was higher at three years of age. The median HV in girls gradually decreased from 7.7 cm at three years to 4.6 cm at seven years. It then remained stationary for one year and then rose to 5.2 cm after ten years. It then slowed down to <2 cm/yr by the age of 14 years. Likewise, the 50th centile HV in boys decreased from 6.1 cm at three years to 4.4 cm at eight years. It then increased to 6.5 cm at 12 years and then fell to <2 cm/yr by the age of 16 years. The pubertal height spurt and pre-pubertal nadir occurred earlier in girls than boys. Boys also had a bigger and longer duration of pubertal spurt.

**Table 2:** Height velocity distribution in boys and girls. HV: height velocity.

Age in years	Boys HV 3rd centile in cm	Boys HV 10th centile in cm	Boys HV 25th centile in cm	Boys HV 50th centile in cm	Boys HV 75th centile in cm	Boys HV 90th centile in cm	Boys HV 97th centile in cm	Girls HV 3rd centile in cm	Girls HV 10th centile in cm	Girls HV 25th centile in cm	Girls HV 50th centile in cm	Girls HV 75th centile in cm	Girls HV 90th centile in cm	Girls HV 97th centile in cm
3-4	3.8	4.2	5.2	6.1	7.7	8.4	10	4.3	5.2	6.3	7.7	8.6	12.1	12.8
4-5	3.7	4.1	4.9	6.1	7.1	8.1	9.4	4.2	4.4	5.3	6	8.1	9.4	10.8
5-6	3.7	4.1	4.8	5.8	6.6	7.7	8.2	4.1	4.2	4.6	4.9	7.5	9.1	10.1
6-7	3.7	4	4.6	5.8	5.9	6.2	8	4	4.1	4.4	4.8	5.5	7.8	9.1
7-8	3.6	4	4.3	4.9	5.5	6.1	7.8	3.9	4.1	4.3	4.6	4.9	6.8	10.3
8-9	3.6	3.9	4.2	4.4	5.2	5.7	8.1	4	4.3	4.4	4.6	5.2	7.8	10.4
9-10	3.6	3.9	4.5	5	5.2	7.2	8.2	3.3	3.6	4	4.7	5.5	8	10.4
10-11	4.4	4.9	5.1	5.2	5.8	7.6	11.7	3.1	3.4	3.6	5.2	7.4	8.9	13
11-12	4.2	4.8	5.2	5.5	6.5	7.8	11.8	1.3	2.3	2.6	4.7	7	7.8	10.4
12-13	3.9	4.4	5.2	6.5	7.8	10.4	13	0.5	1	1.8	3.1	5.2	7.8	10.4
13-14	2.6	3.9	4.9	6.2	7.8	8.9	11.1	0	0.8	1.5	2.6	4.7	6.1	10
14-15	0.6	2.1	2.6	3.9	5.2	8	10.4	0	0.8	1	1.8	3.8	5.5	8.3
15-16	0	0.5	1.3	2.6	3.1	5.7	8.2	0	0.6	1	1.6	3.4	5	5.7
16-17	0	0.5	1	1.8	2.6	4.5	7.8	0	0.1	0.6	1.4	2.6	3.2	4
17-18	0	0.2	1	1.8	2.6	3.4	4.7	0	0	0.4	0.6	0.8	1.3	1.4

## Discussion

This is the first study to report the age- and sex-specific normative data of height velocity in south Indian children between three and 18 years of age. Height velocity percentiles were generated. The data had a good representation of children of either sex and from both urban and rural areas. The mean peak height velocity (PHV) was 7.18±2.56 cm in boys observed at 13 years and 5.8±2.56 cm in girls at 11 years.

One of the earliest longitudinal studies of HV in Indian children was published in 1980 [20], where the height of 303 boys and 260 girls from middle-class families was reassessed at regular intervals over 14 years (1952-1966). PHV was reported at the age of 13.5 years in boys and 12 years in girls. Another longitudinal study on measurements of preschool children during an 18-year study (1965-1966 to 1983-1984) reported PHV at 14 years in boys [21]. Similarly, a study on girls from Northern India reported PHV at 11-13 years old [22]. However, in our study, the PHV was noted at 10-10.9 years and 12-12.9 years in the study from North India, slightly earlier than the studies conducted more than two to three decades ago [20,21,23-25]. This may be due to a secular trend with earlier onset of puberty in Indian children, as has been reported in recent studies [26,27]. However, two recent studies from the western and northern parts of India have reported similar ages for PHV [16,17]. The PHV was noted at 10-10.9 years and 12-12.9 years in the study from North India (Table 3), and at 10.5 years in girls and 13.5 years in boys in the study from Western India (Table 4).

**Table 3:** Mean HV compared to the study. ^a^Dabas et al. [16]. HV: height velocity.

Age in years	Boys HV in cm (mean±SD) in the present study	Boys HV in the study^a^	Girls HV in cm (mean±​​​​​​SD) in the present study	Girls HV in the study^a^
3-4	6.47±1.85	7.36±0.77	7.96±2.60	7.67±1.21
4-5	6.13±1.74	7.18±1.22	6.60±2.17	7.18±1.03
5-6	5.79±1.38	7.23±1.42	6.01±2.09	6.77±1.22
6-7	5.46±1.15	6.92±1.50	5.38±1.68	6.30±1.09
7-8	5.03±1.14	6.47±1.59	5.07±1.49	6.18±1.59
8-9	4.95±1.71	5.84±1.38	5.41±1.95	6.01±1.57
9-10	5.23±1.50	5.71±1.25	5.21±2.07	6.63±1.81
10-11	5.86±1.80	6.08±2.16	5.80±2.56	6.58±1.89
11-12	6.13±1.75	7.07±2.54	5.24±2.78	5.79±2.03
12-13	7.18±2.56	7.82±2.60	3.91±2.61	4.32±2.45
13-14	6.40±2.40	6.67±2.16	3.26±2.62	2.53±2.72
14-15	4.43±2.64	5.46±2.94	2.63±2.29	1.54±1.75
15-16	2.75±2.28	3.18±2.42	2.33±1.70	0.87±1.59
16-17	2.30±2.04	1.71±1.54	1.68±1.36	0.63±1.41
17-18	1.86±1.29	-	0.66±0.43	-

**Table 4:** Median HV compared to the study. ^a^Khadilkar et al. [17]. HV: height velocity.

Age in years	Median HV in cm in boys in the present study	Median HV in cm in boys in the study^a^	Median HV in cm in girls in the present study	Median HV in cm in girls in the study^a^
5.5	5.8	6.5	4.9	6.4
6.5	5.8	6.1	4.8	5.9
7.5	4.9	5.7	4.6	5.7
8.5	4.4	5.4	4.6	5.8
9.5	5	5.2	4.7	6.3
10.5	5.2	5.1	5.2	6.6
11.5	5.5	5.3	4.7	5.9
12.5	6.5	6	3.1	4.2
13.5	6.2	6.8	2.6	2.4
14.5	3.9	6	1.8	1.5
15.5	2.6	4	1.6	0.9
16.5	1.8	2.4	1.4	0.6
17.5	1.8	1.3	0.6	0.3

Unlike the recent studies from western and northern parts of India, our study presents height velocity data for girls and boys between three and five years, albeit derived from a smaller sample size [16,17].

The sexual maturity rating (SMR) of the children, however, could not be done. The availability of SMR data to correlate with the height velocity would have been beneficial in generating curves for early and late maturers separately, thereby helping in generating data that would help in the prediction of age at peak height velocity. However, it is a well-understood fact that assessment of SMR is one of the most difficult tasks performed in population-based studies, as it is less acceptable to parents as well as school authorities and more challenging to the investigators. The availability of parental height values would have helped to look for a correlation with height velocity. An even longer duration of follow-up could have made the results more creditable.

## Conclusions

This is the first large-scale study from South India evaluating height velocity data for children aged three to 18 years. The database for the study was derived from a heterogeneous population, including children from rural and urban areas, thereby representing data from diverse socioeconomic backgrounds. These charts can, therefore, be applied to the economically deprived and privileged alike, because a significant proportion of children catered to by pediatricians and endocrinologists in public hospitals belong to the former group. The generation of HV percentile curves based on longitudinal follow-up of healthy schoolchildren could be a welcome addition to the existing growth reference curves of our population.
